# From Awareness to Action: Co‐Designing Culturally Safe and Equitable Rehabilitation in Canada – Protocol for the Two‐Phase Ireach Qualitative Study

**DOI:** 10.1111/hex.70874

**Published:** 2026-09-17

**Authors:** Nora Bakaa, Lisandra Almeida de Oliveira, Luciana Macedo, Pierre Côté, Fred Toy, Jasdeep Dhir, Yaad Shergill, Astrid DeSouza, Jennifer Ward, Janny Mathieu, Maxi Miciak, André Bussières, Tara Packham, Victor E. Ezeugwu, Jessica J. Wong

**Affiliations:** ^1^ School of Physical Therapy, Faculty of Health Sciences Western University London Ontario Canada; ^2^ Institute for Disability and Rehabilitation Research Ontario Tech University Oshawa Ontario Canada; ^3^ School of Rehabilitation Science, Faculty of Health Science McMaster University Hamilton Ontario Canada; ^4^ Patient Partner Hamilton Ontario Canada; ^5^ The Ottawa Hospital Research Institute Ottawa Ontario Canada; ^6^ University of Manitoba Winnipeg Manitoba Canada; ^7^ Département Chiropratique Université du Québec à Trois‐Rivières Trois‐Rivières Québec Canada; ^8^ Department of Physical Therapy, Faculty of Rehabilitation Medicine University of Alberta Edmaonton Alberta Canada; ^9^ School of Physical and Occupational Therapy, Faculty of Medicine and Health Sciences McGill University Montreal Québec Canada; ^10^ Department of Epidemiology and Biostatistics, Schulich School of Medicine & Dentistry Western University London Ontario Canada; ^11^ Populations and Public Health Research Program, ICES Western London Ontario Canada; ^12^ Institute of Health Policy, Management and Evaluation University of Toronto Toronto Ontario Canada; ^13^ Graduate Studies, Canadian Memorial Chiropractic College North York Ontario Canada

**Keywords:** co‐design, cultural safety, health equity, implementation, patient and public involvement, qualitative research, rehabilitation

## Abstract

**Background:**

Inequities in access, engagement, and outcomes persist across outpatient rehabilitation services in Canada, disproportionately affecting Indigenous Peoples, newcomers and refugees, gender‐ and sexually‐diverse individuals, and people with lower income. Although rehabilitation professionals report strong awareness of equity, diversity, inclusion, and cultural safety principles, a persistent gap remains between knowledge and day‐to‐day clinical practice. Existing resources largely focus on education without providing practical, clinic‐level tools or aligned patient‐facing supports to enable sustained practice change.

**Objective:**

The Inclusive Rehabilitation Equity Assessment and Consensus Hub (iREACH) study aims to co‐design a national, evidence‐informed roadmap of prioritised, actionable strategies to enhance cultural safety and equity in outpatient rehabilitation, including physiotherapy, occupational therapy, and chiropractic settings across Canada.

**Methods:**

The iREACH study will use a two‐phase, sequential co‐design approach. Phase 1 will employ qualitative methods guided by Interpretive Description, including semi‐structured interviews, to explore patients' experiences of cultural safety and unsafety in rehabilitation care. Participants will be purposefully sampled to reflect diversity across social identities and locations, geographic regions, and provider types. Data will be analysed using inductive Reflexive Thematic Analysis within the overarching framework of Interpretative Description. Phase 2 will involve a national co‐design workshop with an intersectional panel of patients, clinicians, and system leaders. Findings from Phase 1 and prior research will be collaboratively translated into prioritised strategies and practical clinician‐ and patient‐facing resources to support culturally safe and equitable rehabilitation care.

**Expected Outcomes:**

The primary outcome will be a bilingual (English and French) co‐designed Shared Blueprint comprising prioritised, actionable strategies for clinicians, organisations, and health system leaders, together with complementary patient‐facing resources to support culturally safe and equitable rehabilitation care across Canada. By integrating lived experience, clinical perspectives, and system leadership, iREACH aims to bridge the gap between cultural safety intent and implementation, supporting more equitable, accessible, and patient‐centred rehabilitation care across Canada.

**Patient or Public Contribution:**

Patient partner has been actively involved in the study design, including development of the research questions, interview guide, and co‐design approach. They will remain involved throughout the study as member of the research team contributing to data interpretation and the co‐development of study outputs.

## 1 Introduction

When rehabilitationis experienced as culturally unsafe, trust and engagement in care often deteriorate before treatment can meaningfully begin. For many individuals, particularly those from equity‐deserving groups in Canada, including Indigenous Peoples, newcomers and refugees, gender‐ and sexually‐diverse individuals, and people with lower income–experiences of unsafe care can shape whether care is initiated, continued, or trusted. Missed appointments, early dropouts, and uncompleted treatment plans disproportionately affect these populations [1, 2, 3, 4]. These patterns contribute to avoidable limitations in functioning, increased healthcare utilisation, and the widening of health inequities across rehabilitation systems [3, 5, 6, 7]. Evidence consistently demonstrates that inequities in rehabilitation are not solely driven by clinical need, but also by structural, cultural, and organisational barriers that shape how care is accessed, delivered, and sustained [8, 9]. For the purpose of this study, outpatient rehabilitation refers to privately funded physiotherapy, occupational therapy, and chiropractic services delivered in community‐based settings.

Over the past decade, increasing attention has been directed toward cultural safety, equity, and inclusion within professions involved in delivering rehabilitation. Three national surveys conducted by our group among physiotherapists [10, 11], chiropractors [12, 13], and occupational therapists [14] suggest a consistent pattern: while clinicians report high levels of awareness and sensitivity related to cultural competence, they experience considerable challenges translating these principles into everyday clinical practice. Across professions, clinicians describe uncertainty about how to adapt assessments and treatment plans in culturally responsive ways, alongside structural constraints such as cost, language discordance, and time pressure as key barriers to delivering equitable care [10, 12, 14]. Workforce data further highlight the under‐representation of Black, Indigenous, and Latinx clinicians across professions involved in rehabilitation, compounding access barriers for communities who already face structural marginalisation [11, 13, 14]. Together, these findings reveal that a possible persistent gap exist between intention and action, where clinicians value safety but lack practical means to implement it consistently in real‐world settings.

Qualitative interview studies conducted by our group with physiotherapists, chiropractors, and occupational therapists [15, 16] further highlighted why this gap appears to persists. Across all three professions (chiropractic, physiotherapy and occupational therapy), clinicians consistently emphasised that theoretical knowledge alone is insufficient to support meaningful practice change. Instead, they described a need for clinic‐level supports that can be integrated into everyday care. These include access to translated intake forms, clear pathways to access professional interpreters, brief and accessible training resources, and flexible care models to support patients facing financial barriers [12]. Across professions, clinicians also highlighted uncertainty in how to adapt rehabilitation approaches in culturally responsive ways, alongside a lack of concrete examples and patient‐facing resources to guide practice [10, 14]. Importantly, professionals with prior equity, diversity, and inclusion (EDI) training reported higher levels of cultural competence, yet continued to call for training that is concise, practice‐oriented, and aligned with the realities of busy clinical environments [10, 12]. Together, these findings suggest that clinicians may not seek additional conceptual frameworks but rather actionable, clinic‐level tools supporting the translation of cultural safety principles into routine practice. The present study builds upon this established programme of research by co‐designing practical, evidence‐informed resources to support the implementation of cultural safe rehabilitation practice.

However, existing cultural‐safety resources and toolkits in rehabilitation remain limited and predominantly provider‐focused, with insufficient attention to patient‐facing materials and implementation sustainability. A recent environmental scan of Indigenous cultural‐safety initiatives [17] in Ontario identified 15 training programmes and organisational guides; however, these resources were predominantly provider‐focused, with limited inclusion of patient‐facing materials and minimal evaluation of whether increased knowledge translated into sustained changes in practice. Only one programme incorporated follow‐up metrics to assess implementation, and most lacked mechanisms for accountability or long‐term sustainability. Similar patterns have been observed internationally. A recent review [18] examining cultural‐safety initiatives in Australia, New Zealand, and Canada, suggested that meaningful and sustained improvements in culturally safe care are most likely when education is combined with organisational system‐level supports, such as dedicated roles (e.g., Indigenous liaison officers or cultural brokers), leadership endorsement, and policy‐level change. Despite this, many available resources continue to rely on one‐time educational approaches that appear to be insufficient to support behaviour change in complex, real‐world rehabilitation environments.

Despite increasing recognition of cultural safety within rehabilitation professions, important gaps remain in understanding how culturally safe and unsafe care is experienced by patients navigating rehabilitation systems [19, 20, 21, 22, 23, 24]. Existing rehabilitation research has primarily focused on specific populations, healthcare contexts, clinician perspectives, and organisational approaches, with relatively limited primary qualitative evidence describing patients' experiences across rehabilitation services [23, 24]. Furthermore, within the Canadian outpatient rehabilitation context, the available literature offers limited insight into how structural inequities, interpersonal interactions, and contextual barriers shape patients' trust, engagement, and continuity of care. These gaps are particularly important for individuals from equity‐deserving groups, whose experiences of healthcare marginalisation may not be fully reflected in provider‐oriented research or implementation initiatives [25, 26]. Exploring these lived experiences is essential to informing the co‐design of culturally safe, equitable, and practice‐relevant rehabilitation resources across Canada.

Together, this body of evidence highlights a critical mismatch between existing cultural‐safety resources and the practical needs of clinicians and patients in the context of rehabilitation. Current resources prioritise knowledge acquisition, while offering limited support for implementation in routine care. This gap underscores the need for concise, integrated, action‐oriented resources combining clinician‐facing tools with aligned patient‐facing supports, and that are developed in partnership with those directly impacted by inequities in rehabilitation care.

In response to these gaps, we designed the Inclusive Rehabilitation Equity Assessment and Consensus Hub (iREACH) study to co‐develop a national, evidence‐informed roadmap to support culturally safe rehabilitation practice. By centring lived experience alongside clinical and system perspectives, this study seeks to move beyond awareness toward meaningful and sustainable practice change. The present study represents one step in a broader programme of research. The co‐designed Shared Blueprint developed through iREACH will subsequently inform the development of implementation outcome measures, followed by feasibility testing in clinical settings and, ultimately, evaluation through a randomised controlled trial.

### 1.1 Study Objectives

The iREACH study aims to co‐design a national evidence‐informed roadmap (the Shared Blueprint) to enhance cultural safety and equity within outpatient rehabilitation services across Canada, with a focus on privately funded physiotherapy, chiropractic, and occupational therapy services delivered in community‐based settings. The study will comprise two sequential and interconnected phases, each with specific objectives:

***Phase 1: Generate the evidence base***: To explore and describe patients' experiences of cultural safety among a diverse cohort of individuals, including Indigenous Peoples, newcomers, gender‐ and sexually‐diverse individuals, and Canadians with lower income, who have used or discontinued rehabilitation services (i.e., physiotherapy, chiropractic, or occupational therapy).

a. To identify clinic‐level factors, behaviours, environments, and policies that make rehabilitation care feel culturally safe or unsafe to patients accessing rehabilitation services.
b. To capture variations in patients' experiences across provinces, provider types, and social identities (e.g., Indigenous identity, race, ethnicity, gender, sexuality, income, rurality, and newcomer status).

**Phase 2**: ***Co‐design framework and workshop**:* To collaboratively translate the qualitative findings from Phase 1, alongside previous research, into actionable, equity‐oriented strategies, through a structured co‐design process. This phase will bring together an intentionally balanced, intersectional panel, comprising patients with lived experience, clinicians, and policymakers, who will jointly refine and prioritise clinician‐facing and patient‐facing strategies.

a. To synthesise patient interview findings with previous clinician interview data to draft a preliminary list of actions.
b. To develop and prioritise clinician‐facing and patient‐facing strategies to support culturally safe and equitable rehabilitation care.

## 2 Methods

### 2.1 Study Design

**Figure 1:**
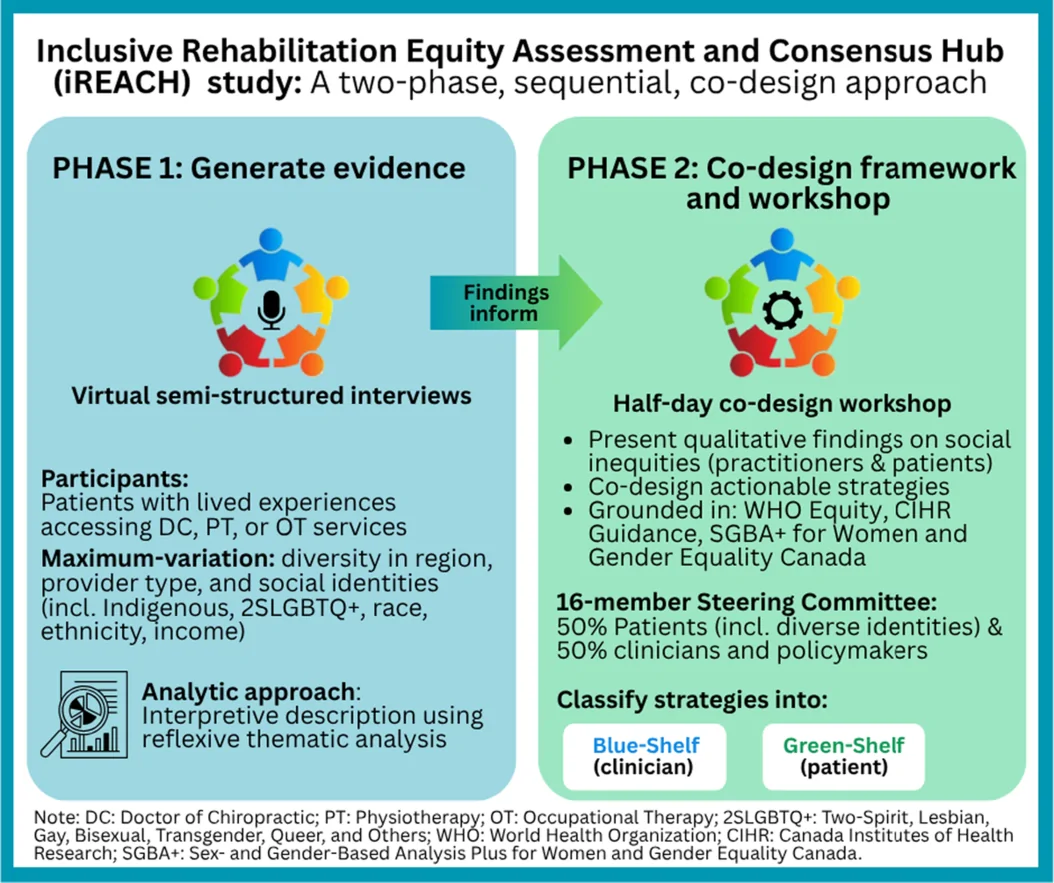
Overview of the iREACH study design.

This study will employ a two‐phase, sequential co‐design approach (Figure 1). This approach was selected to ensure that the development of the proposed resources is grounded in patients' lived experiences and informed by clinicians' and knowledge users' perspectives,thereby supporting the creation of contextually relevant and implementable solutions. Phase 1 will use a qualitative methodology to explore how cultural safety is experienced within rehabilitation, generating an evidence base to inform co‐design (Phase 2). Phase 1 will be guided by Interpretive Description [27] as the overarching qualitative approach, incorporating semi‐structured interviews, with data analysed using Reflexive Thematic Analysis. Phase 2 will engage patients, clinicians, and health system leaders in collaboratively developing actionable, clinic‐level strategies and tools to enhance culturally safe rehabilitation practice. Together, these two phases move from experience‐based understanding to the collaborative development of practical, evidence‐informed resources in partnership with patients, clinicians, and system knowledge users. The study is limited to privately funded outpatient rehabilitation settings because models of care delivery and implementation differ substantially between publicly funded and private practice environments in Canada. However, the resources developed for this study may be adapted in future stages to different contexts. This study will be reported in accordance with the Consolidated Criteria for Reporting Qualitative Research (COREQ) [28] for the qualitative components, alongside the Guidance for Reporting Involvement of Patients and the Public (GRIPP2) [29] to ensure transparent reporting of the co‐design process.

***Phase 1: Generate the Evidence Base***

#### 2.1.1 Participants and Sampling

We aim to recruit up to 60 individuals with lived experience of rehabilitation services in Canada within the past 2 years (e.g., physiotherapy, chiropractic, or occupational therapy), or until data sufficiency is achieved [30]. A larger sample is anticipated to support maximum variation sampling across intersecting social identities, experiences of rehabilitation care, and geographic regions, ensuring adequate representation of diverse lived experiences across the rehabilitation context across Canada.

Individuals will be eligible to participate if they are ≥ 18 years of age, have received or discontinued outpatient rehabilitation in the last 24 months, and are fluent in English or French. A maximum variation sampling strategy will be used to intentionally recruit participants with a diverse lived experiences and intersecting social identities across the outpatient rehabilitation context [31]. Sampling will be structured to reflect diversity across (a) province or region (including rural and urban settings), (b) provider type (physiotherapy, chiropractic, or occupational therapy), (c) rehabilitation programme completion status (completed vs. exited rehabilitation early) (d) age and gender identity and sexual orientation, (e) Indigenous identity and community affiliation, (f) race and ethnicity, (g) newcomer and refugee status, (h) income and education level, and (i) religion or spiritual affiliation. In addition, snowball sampling will be used as a complementary recruitment strategy, particularly to support engagement with Indigenous participants and other communities where recruitment may be facilitated through trusted networks [31].

Recruitment will occur pan‐nationally through professional associations and networks, patient networks, social media, and community partners (e.g., Indigenous friendship centres, newcomer agencies, 2SLGBTQ+ organisations). Study information and recruitment materials (e.g., posters) will be disseminated through these channels to raise awareness of the study. Recruitment will be conducted through organisational emails and social media advertisements. After informed consent is obtained, participants will be invited to complete study procedures, including a demographic survey and an individual qualitative interview conducted virtually.

### 2.2 Data Collection Procedures

Demographic information (e.g., age, gender identity, racial or ethnic identity, geographic region, and rehabilitation care experiences) will be collected using an online questionnaire (Qualtrics, Provo, UT [32]), accessed through a link provided to participants prior to the interview. Participants will receive the interview guide in advance of the interview to allow time for reflection before the discussion.

In Phase 1, data will be collected through individual semi‐structured interviews, guided by Interpretive Description, and conducted virtually via a secure videoconferencing platform (Western University's institutionally licensed Zoom platform) [33]. Participants who prefer not to use videoconferencing may join the interview via telephone using the dial‐in option available through Western University's licensed Zoom platform. At the conclusion of each interview, the interviewer will conduct a brief member‐checking exercise by summarising the participant's key experiences and preliminary interpretations and inviting the participant to confirm, clarify, or expand upon these points before the interview concludes.

Interpretive Methodology: Interpretative description is a qualitative methodological approach designed to generate clinically meaningful knowledge grounded in participants' experiences within specific health care contexts [27]. This approach is particularly suited to applied health research, as it emphasises understanding patterns, meanings, and variations in human experience that can directly inform practice, rather than producing abstract theory or statistically generalisable findings. Consistent with this methodology, the interviews aim to develop an in‐depth understanding of how participants experience inclusion, exclusion, communication, respect, and the clinic environment within rehabilitation settings, centreing their perspectives to inform practice‐relevant insights. Members of our research team (LA and JM, for interviews in English and French, respectively) will conduct one‐on‐one (45‐60 min) interviews, using a semi‐structured interview guide (Appendix 1). The interview guide was developed based on prior research on cultural safety, humility, equity‐oriented care, and health justice, alongside input from the research team, patient partner, and collaborating health organisations (e.g., the Canadian Physiotherapy Association and Canadian Chiropractic Association). Interviewers will adopt a participant‐centred, flexible interviewing approach, allowing participants to determine the depth and direction of discussion according to their comfort level.

All sessions will begin with a review of consent and confidentiality, and voluntary participation. Interviews will be audio‐recorded and transcribed verbatim. Data collection and analysis will occur concurrently and iteratively, with ongoing analysis informing subsequent data collection, including refinement of the interview guide and sampling decisions as new topics emerge. Data collection will continue until data sufficiency is achieved, defined as the point at which sufficient depth, richness, and diversity of perspectives are obtained to support a robust and meaningful interpretive analysis aligned with the study objectives [30].

Participants may experience emotional discomfort when discussing their experiences. To support participants, information about relevant support resources will be included in the Letter of Information and Consent. During and/or following the interview or group session, participants will be reminded that these resources are available. During the virtual sessions, this information will also be shared via the chat function or follow‐up communication to ensure participants can access it easily.

### 2.3 Data Analysis

Data will be analysed using an inductive Reflexive Thematic Analysis [34] within the overarching framework of Interpretative Description [27]. This analytic approach is aligned with the applied aim of generating practice‐relevant insights to inform culturally safe rehabilitation care, while remaining responsive to the complexity and diversity of participants' lived experiences. Insights gained through the member‐checking process will be considered during coding and interpretation to ensure that the analysis accurately reflects participants' intended meanings. Emerging themes will also be discussed with our patient partner throughout the analytic process to support interpretation and ensure that the findings remain grounded in participants' lived experiences.

All interviews transcripts will be managed and analysed using NVivo software [35]. Reflexive thematic analysis [25] will begin with a close, line‐by‐line reading of transcripts to support familiarisation with the data and the identification of initial analytic insights. Two analysts will each code the first set of transcripts, generating an enriched preliminary thematic and code structure and analytic memos to capture emerging interpretations. These codes will be discussed collaboratively with additional team members to develop a living code record, which will iteratively be refined as new data are incorporated and analytic understanding deepens.

The analytic process described above will proceed iteratively in alignment with the six phases of reflexive thematic analysis described by Braun and Clarke [34]. (1) familiarisation with the data through repeated reading, (2) generation of initial codes, (3) searching for patterns and potential themes across dataset, (4) reviewing and refining themes in relation to coded extracts and the full dataset, (5) defining and naming themes, and (6) producing the analytic narrative. Throughout the analytic process, ongoing reflexive dialogue within the research team will be used to critically examine assumptions, challenge interpretations, and support the development of meaningful, contextually grounded insights. Additionally, throughout the analytic process, attention will also be given to how intersecting social identities and structural contexts shape participants' experiences across equity‐deserving groups.

### 2.4 Trustworthiness and Rigour

Trustworthiness and rigour will be supported through a combination of complementary strategies. Analytic rigour will be supported through iterative data collection and analysis, reflexive thematic analysis methods, and ongoing update of the coding record as analytic understanding develops. Reflexivity will be maintained through analytic memo‐writing and regular team debriefs to critically examine assumptions, positionality, and interpretive decisions. Credibility will be strengthened through team‐based analysis involving researchers from diverse professional backgrounds, identities, geographic contexts, and lived experiences, allowing multiple perspectives to inform interpretation. To support the richness and depth of the data, sampling will be guided by maximum variation and intersectional stratification, and data collection will continue until data sufficiency is achieved. Transparency and auditability will be enhanced through systematic documentation of analytic decisions and the use of qualitative data management software. Participants in Phase 1 will receive a CAD $50 honorarium in recognition of their time and contribution to the study.

***Phase 2: Co‐Design Framework And Workshop***

### 2.5 Participants and Sampling

At the end of the Phase 1 interview, participants will be invited to express interest in remaining involved as decision‐makers in Phase 2. From these, approximately eight participants with lived experience will be purposefully selected using an intersectional lens to ensure representation across gender and sexual diversity, Indigeneity, racialized perspectives, newcomer and refugee backgrounds, rural and urban settings, age, and income levels.

These eight participants will join five clinicians (representing physiotherapists, chiropractors, and occupational therapists) and three health system leaders (one per profession), resulting in a 16‐member panel, composed of approximately 50% patients and community members and 50% clinicians and policymakers. If representation gaps are identified (e.g., low engagement from specific equity‐deserving groups), additional community members will be invited to ensure diversity and balance.

### 2.6 Data Collection Procedures

In Phase 2, clinicians and health system leaders participating in the co‐design workshop will be asked to complete a brief online demographic questionnaire (via Qualtrics) prior to the workshop to describe their professional background (e.g., professional role, years of experience, etc). Patient participants will not be asked to complete an additional demographic questionnaire in Phase 2, as this information will have already been collected during Phase 1. The virtual co‐design workshop will be guided by an equity‐oriented facilitation approach. The workshop will be informed by established equity‐focused frameworks, including the World Health Organization (WHO) Equity Framework, the Canadian Institutes of Health Research (CIHR) Guidance on Sex, Gender, and Health Research [36], and the Sex‐ and Gender‐Based Analysis Plus (SGBA +) framework of Women and Gender Equality Canada [37]. The facilitation will also draw on Experience‐Based Co‐Design (EBCD) [38] and the Health Equity Implementation Framework (HEIF) [39] to ensure inclusive and culturally safe participation.

To maximise time for interactive discussion, the workshop will be delivered through a combination of asynchronous and synchronous activities. Prior to the live workshop, participants will receive a secure link to a pre‐recorded presentation summarising the Phase 1 findings, previous published qualitative results on social inequities among rehabilitation practitioners, together with instructions to review the material before attending the session. At the beginning of the live workshop, facilitators will provide a brief summary of the findings and answer participants' questions before proceeding to the co‐design activities.

The live workshop will focus on collaboratively developing and refining actionable strategies to address the barriers identified in Phase 1 and previous published studies. Final prioritation of the proposed actions will occur asynchronously following the workshop. Conducting the prioritisation asynchronously will allow the research team to consolidate and organise the recommendations generated during the workshop before participants complete the final ranking exercise.

During the breakout discussions of the segment 2, facilitators will use a structured facilitation approach combining the 1‐2‐4‐All Liberating Structure and principles of the Nominal Group Technique to promote equitable participation and support the development of practical, actionable recommendations [40, 41]. Participants will first reflect individually, then discuss their ideas in pairs, followed by groups of four, before engaging in the full breakout group discussion. Facilitators will then guide participants through a strusctured process to share, clarify, discuss, and consolidate proposed actions/strategies. Final prioritisation will occur following the workshop through an asynchronous online survey.

Following the workshop, participants will receive an emailed link to an online survey to independently prioritise the proposed actions/strategies. Conducting the prioritisation asynchronously will allow the research team to consolidate overlapping recommendations and refine their wording for clarity, while preserving participants' intended meaning, before the final prioritisation exercise. For each proposed action, participants will indicate the extent to which they agree that the action should be included in its respective Blue Shelf (i.e., recommendations for clinicians, organisations, and health system leaders to support culturally safe rehabilitation practice) or Green Shelf (i.e., patient‐facing resources, such as educational materials) category, considering its importance, potential equity impact, feasibility, and acceptability, using a 5‐point Likert‐type scale ranging from 1 (strongly disagree) to 5 (strongly agree). Each participant's rating will carry equal weight, irrespective of knowledge user group.

To support participation from both English‐ and French‐speaking participants, the workshop will be conducted as a single session using Western University's Zoom platform. Participants will be able to select captions in either English or French through Zoom's live translation feature to support communication during the workshop. This feature allows participants to engage in the discussion in their preferred language by viewing translated captions during the session. Participants will receive step‐by‐step instructions in their preferred language in advance on how to enable and use this feature (e.g., selecting their preferred language for captions) to facilitate full participation. Workshop discussions and facilitation will be conducted in English and French only. Member of the research team who is proficient in both English and French (P.C. and J. M.) will be present during the workshop to assist participants as needed and to support communication throughout the session. The facilitation team will actively monitor communication and engagement throughout the session to ensure that participants are able to meaningfully contribute to group discussions and activities.

The workshop will include three main segments (Table 1)

**Table 1:** Structure and key activities of the phase 2 co‐design workshop.

Segment	Purpose	Key activities	
1. Asynchronous review of findings	To establish a shared understanding of existing evidence and emerging findings related to inequities in rehabilitation	Participants receive a secure link to a pre‐recorded video presentation summarising: − Previous published qualitative results on social inequities among rehabilitation practitioners [9, 27]. − Emerging Phase 1 findings capturing patient perspectives on cultural safety and unsafety.	
2. Rotating small‐group breakout discussions	To co‐develop actionable strategies and practical tools to address inequities identified in qualitative findings	− Welcome, land acknowledgement, and introductions. − Brief recap on the asynchronous review of findings − Short question‐and‐answer to clarify key messages. − Participants will be divided into small mixed groups (4–6 people per group) that intentionally combine patients, clinicians, and system leaders. − To ensure equitable participation and reduce power imbalances, participants will first reflect individually, then discuss ideas in pairs, followed by groups of four, before engaging in the full breakout discussion. − Each group will rotate through a series of Western Zoom breakout rooms, with each room focused on some prespecified theme derived from qualitative findings on inequities from both patients′ and practitioners′ perspectives. − Facilitators will guide participants through a Nominal Group Technique, including silent idea generation, round‐robin sharing, structured discussion, and collaborative refinement of proposed actions. − A rapporteur within each group will take structured notes during every rotation. − By the end of this segment, each group will have contributed to discussions across all themes, generating a comprehensive list of proposed actions.	
3. Plenary synthesis and prioritisation	To synthesise, refine, and prioritise proposed strategies based on feasibility and potential impact	− The full group will reconvene for collective synthesis, moderated by a member of our research team. − The moderator will guide the presentation of summaries of each group′s insights, theme‐by‐theme: for each theme, rapporteurs (and other participants as desired) will share suggestions and discuss the proposed strategies collectively. − After all themes have been discussed, the moderator will facilitate a final dialogue to refine and consolidate the list of actions. − All proposed strategies will then be collaboratively categorised as Blue Shelf (clinician‐facing) or Green Shelf (patient‐facing). − Participants subsequently receive an emailed survey to independently rank the proposed actions according to feasibility and potential impact.

The synchronous segments of the workshop will be audio‐recorded and transcribed verbatim. Chat logs and field notes will be retained as Supporting material to support the analytic process. At the time of protocol submission, funding to compensate Phase 2 workshop participants had not yet been secured.

### 2.7 Data Analysis

Workshop data (transcripts, rapporteur notes, and chat logs) will be analysed using a qualitative descriptive synthesis approach, consistent with applied health research and co‐design principles. First, all proposed actions will be compiled and organised around the major themes emerging from Phase 1 and from previously published studies on health inequity from the partitioners' perspective [10, 16]. Two researchers will independently review the material, merging overlapping ideas and refining wording to ensure clarity while preserving participants' intended meaning. The resulting actions will be categorised as Blue Shelf or Green Shelf and presented to participants in the post‐workshop priorization survey. The Blue Shelf will consist of recommendations for clinicians, organisations, and health system leaders to support culturally safe rehabilitation practice. The Green Shelf will consist of patient‐facing resources (e.g., educational material) designed to support patients in navigating rehabilitation services and engaging in culturally safe care.

Quantitative data from post‐workshop prioritisation survey will be summarised descriptively. For each proposed action, the proportion of participants selecting each response category will be calculated. Consensus for inclusion in the Blue Shelf or Green Shelf will be defined a priori as ≥ 75% of respondents rating an action as 4 (agree) or 5 (strongly agree) [42, 43]. Actions reaching this threshold will be considered to have achieved consensus for inclusion. Actions not reaching the consensus threshold will not be automatically excluded; rather, patterns of agreement and disagreement will be examined alongside the qualitative workshop data to identify potential differences in perspectives across knowledge user groups and equity considerations. Particular attention will be given to recommendations reflecting the priorities of equity‐deserving groups whose perspectives could be obscured by aggregate ratings.

The team will conduct reflexive debriefs with patient partners to verify inclusiveness, cultural congruence, and alignment with participants' intent. The final output will be a bilingual preliminary Shared Blueprint Beta, representing a collaboratively developed, structured set of prioritised recommendations for clinicians, organisations, and health system leaders (Blue Shelf), together with complementary patient‐facing resources (Green Shelf), to enhance cultural safety in rehabilitation practice. In addition, dissemination will include tailored knowledge translation approaches to support accessibility and relevance across diverse participant groups, including Indigenous participants, newcomers, and other equity‐deserving populations. This may include plain‐language summaries and culturally appropriate materials (e.g., visual formats such as infographics).

### 2.8 Researcher Characteristics and Reflexivity

The iREACH research team is intentionally composed to reflect diverse identities, lived experiences, and professional backgrounds aligned with the populations this work aims to serve. Dr. Nora Bakaa (Ontario), a Muslim former refugee and person of colour, brings perspectives shaped by racialization and migration. Dr. Luciana Macedo (Ontario), a Latinx clinical‐trialist and physiotherapist, contributes insights informed by her immigration journey and experiences with language and settlement barriers. Dr. Jessica Wong (Ontario), an Asian Canadian health‐services researcher, ensures our MSK systems approach remains grounded in the realities of racialized communities. Dr. Pierre Côté (Ontario), a French‐Canadian epidemiologist, strengthens our population‐health lens and supports linguistic and regional inclusivity. Dr. Astrid DeSouza (Ontario), is a South Asian Canadian public health researcher who brings her scholarly training in Indigenous health research, in particular pain‐related disabilities. Mr. Fred Toy is a patient‐engaged researcher with firsthand experience of musculoskeletal conditions. Ms. Jasdeep Dhir (Ontario), a physiotherapist and researcher of colour, and Dr. Yaad Shergill (Ontario), a racialized chiropractor and co‐lead of the Power Over Pain Portal, bring frontline perspectives on access and culturally safe rehabilitation. Dr. Jennifer Ward (Manitoba), a Mi'kmaq chiropractor and PhD candidate, embeds Indigenous ways of knowing and safeguards adherence to OCAP® and relational‐accountability principles. Dr. Tara Packham (Ontario), an occupational therapist with deep clinical experience in outpatient rehabilitation and researcher, provides expertise in knowledge translation and implementation for clinical practice. Dr. Janny Mathieu (Québec), a chiropractor and PhD candidate, adds Francophone and Québec health‐system perspectives, along with experience working with underserved communities. Dr. Maxi Miciak (Alberta), a queer rehabilitation researcher, brings expertise in developing and implementing EDI impact frameworks. Dr. André Bussières (Québec), a French‐Canadian health systems and mixed‐methods researcher, brings expertise in population health, consensus methods, and Indigenous health research. Dr. Victor Ezeugwu (Alberta), an African‐Canadian physical therapist and assistant professor, offers expertise in immigrant experiences, 24‐h movement behaviours, and rehabilitation outcomes. Dr. Lisandra Almeida de Oliveira (Ontario), a Latina physiotherapist and rehabilitation scientist, brings lived experience as an immigrant and expertise in culturally responsive rehabilitation research. Together, the team's diverse positionalities and experiences are actively engaged throughout the research process to support reflexive practice, challenge assumptions, and ensure that the co‐designed outputs remain grounded in equity, inclusivity, and cultural safety.

### 2.9 Limitations

This study is subject to some methodological and contextual limitations. Although sampling will be intentionally diverse, it may not fully capture the breadth of intersecting experiences across Canada's rehabilitation landscape. Furthermore, the heterogeneity of participants and rehabilitation contexts may limit the extent to which the resulting recommendations fully address the specific needs of every population, geographic region, or practice setting. While the co‐design process aims to develop broadly relevant and actionable resources, further contextual adaptation may be needed to support implementation in specific settings. Furthermore, as interviews rely on participants' retrospective accounts, findings may be influenced by challenges with recall. Participation will be limited to individuals able to participate in English or French due to available study resources and feasibility constraints, which may limit participation among some individuals and communities who experience language‐related barriers in accessing rehabilitation care. In addition, conducting interviews and workshops virtually may limit participation among individuals with restricted access to technology or lower digital literacy, which may disproportionately affect equity‐deserving groups. While efforts will be made to mitigate these barriers (e.g., offering phone‐based participation, providing technological support for virtual sessions), some degree of exclusion may remain.

## Author Contributions

**Nora Bakaa:** Conceptualization; Funding acquisition; Writing – original draft; Investigation; Methodology. **Lisandra Almeida de Oliveira:** Writing – original draft; Project administration. **Luciana Macedo:** Conceptualization; Investigation; Writing – review and editing. **Pierre Côté:** Investigation; Conceptualization; Writing – review and editing. **Fred Toy:** Writing – review and editing. **Jasdeep Dhir:** Writing – review and editing. **Yaad Shergill:** Writing – review and editing. **Astrid DeSouza:** Writing – review and editing; Methodology. **Jennifer Ward:** Writing – review and editing; Methodology. **Janny Mathieu:** Writing – review and editing. **Maxi Miciak:** Writing – review and editing. **André Bussières:** Writing – review and editing. **Tara Packham:** Writing – review and editing. **Victor E Ezeugwu:** Writing – review and editing. **Jessica J Wong:** Writing – review and editing; Conceptualization; Investigation; Funding acquisition; Supervision.

## Ethics Statement

Ethical approval for this study was obtained from the Western University Health Sciences Research Ethics Board (HSREB) on August 24, 2026 (Review Reference: 2026‐128707‐125812; Project ID: 128707). The project's patient partner (FT) has been actively involved in the design of this study, including the development of the research questions, interview guide, and co‐design approach. They will continue to contribute throughout the study by supporting the interpretation of Phase 1 findings and participating in the co‐design activities in Phase 2 as a member of the research team. The patient partner will not be enroled as a study participant, and their contributions will be documented through research team discussions and meeting records and will inform study design, interpretation and co‐design activities. These contributions will not be treated as participant data in the analysis.

## Conflicts of Interest

The authors declare no conflicts of interests.

## Patient and Public Involvement Statement

The project's patient partner (FT) has been actively involved in the design of this study, including the development of the research questions, interview guide, and co‐design approach. They will continue to contribute throughout the study by supporting the interpretation of Phase 1 findings and participating in the co‐design activities in Phase 2 as a member of the research team. The patient partner will not be enrolled as a study participant, and their contributions will be documented through research team discussions and meeting records and will inform study design, interpretation and co‐design activities. These contributions will not be treated as participant data in the analysis.

## Supporting information


Supporting File

## Data Availability

The datasets generated and/or analysed during the current study will not be publicly available due to the sensitive nature of the data and the need to protect participant confidentiality but may be available from the corresponding author on reasonable request and with appropriate ethical approvals.
