# Supplementary material for: From Awareness to Action: Co‐Designing Culturally Safe and Equitable Rehabilitation in Canada – Protocol for the Two‐Phase Ireach Qualitative Study
Source: Health Expect. 2026 Sep 17;29(5):e70874. doi: 10.1111/hex.70874 (PMC13583488; doi:10.1111/hex.70874)
Supplement: Supplementary file 1 [file HEX-29-e70874-s001.docx]

**Appendix 1:** Semi-Structured Interview (Phase 1)

Hi [participant’s name],

My name is ______________, and I’m _____________________________________ with the iREACH project. Thank you so much for taking the time to meet with me today and for agreeing to share your experiences.

As we discussed earlier, this study aims to understand how people experience rehabilitation care in Canada, and what makes those experiences feel safe, respectful, and/or inclusive – or at times, less so. What you share today will help us identify ways to make rehabilitation more culturally safe and equitable for everyone.

Before we begin, I’d like to confirm a few details:

- Have you received and had a chance to review the consent form?
- Do you have any questions about the study or your participation?
- Are you comfortable proceeding with the interview today?

With your permission, I will be recording our conversation using Zoom, which will also include a transcript of our conversation, so that I don’t miss anything you share. The recording will be stored securely and deleted once the transcript has been verified for accuracy. Is that okay with you?

Thank you! I’ll start the recording now.

Some of the questions today may bring up emotions and difficult memories. You’re welcome to take a break, pause, skip any question, or stop the interview at any time. If it’s easier, you can also signal that you need a break by raising your hand. Just let me know what you prefer.

Section 1: Introductions and Context

1. To start, could you tell me a little about yourself - whatever you’re comfortable sharing - such as the kind of rehabilitation services you’ve used and when that was? What led you to seek rehabilitation at that time? If you’re not sure whether a service fits within rehabilitation as discussed here, feel free to mention it.
2. Before we go any further, when you hear the term “rehabilitation”, what does it mean to you?
   a. Where does that understanding come from?
   b. Has it changed over time?

Before we continue, when I say ‘rehabilitation,’ I’m using it broadly. I mean services that support people in improving or maintaining physical, mental, or social functioning - such as help with daily activities, work, mobility, or participation in social life. This could include services like physiotherapy, chiropractic care, occupational therapy, exercise programs, pain management programs, cognitive behavioural therapy, or other similar supports, whether provided in clinics, hospitals, or the community.

3. Expectations: Thinking back to when you first started rehabilitation, what were you hoping the experience would be like?
a) Access to care (e.g., how easy or difficult it would be to get an appointment)
b) First points of contact or referral pathways
c) Interactions and communication with health care providers
d) Type and content of care provided
e) Follow-up, continuity, or coordination of care
f) Any concerns, worries, or hesitations you had about attending rehabilitation or about the rehabilitation professional you were going to see?

Section 2: Overall Experience of Care

4. Can you walk me through what a typical appointment or visit was like for you?
a) About how long did you receive rehabilitation services?
b) Where did you receive care (e.g., type of clinic or setting)?
c) How did you decide where to seek care?
d) What was the main health concern or condition you were seeking care for, if you feel comfortable sharing?
5. What aspects of care or interaction felt positive or supportive?
6. Were there any moments during your care that felt uncomfortable or unsafe? If so, can you tell me about them?

Section 3: System and Access Factors

Access to healthcare services and the way systems are organized can shape people’s experiences of inclusion, exclusion, and belonging. We’d now like to talk about how you navigated the healthcare system.

7. What was your experience of navigating the healthcare system to access rehabilitation services?
a) Were there any barriers, such as wait times, cost, transportation, or eligibility? If yes, please describe.
b) Did these experiences affect your sense of inclusion, fairness, or belonging? If so, how?
8. Once you were able to access a clinic or service, how did organizational policies (for example, intake forms, scheduling, or communication) affect your experience of inclusion or exclusion?
9. Were there any supports, resources, or people who helped you navigate the system? If yes, please describe.
10. How did having (or not having) these supports affect your experience?

Section 4: Communication and Respect

11. How did your rehabilitation provider communicate with you? In what ways, if any, did you feel heard and understood?
12. Were there any language, cultural, or identity-related barriers that affected how you communicated?
13. Can you recall a time when you felt particularly respected or disrespected by a provider or the clinic environment?
14. Did you feel like you were “in the driver’s seat” during your interactions? Did you feel like you had options, choices, and the power to choose?
15. Did you feel like your family or support systems were welcomed to participate in rehabilitation if you requested this?

Section 5: Inclusion, Belonging, and Cultural Safety

16. What does ‘feeling culturally safe’ mean to you in a healthcare or rehabilitation setting?
17. Did you ever feel that aspects of your background (such as your language, gender, ethnicity, staff diversity, and physical space) influenced your comfort level during your care? If yes, how?
18. Did the clinic’s environment (such as signage, accessibility, staff diversity, or physical space) influence your comfort level or sense of safety? If so, how?
19. Were there any assumptions made about you (positive or negative) that shaped your experience? If so, can you tell me about them?
a) Did you feel that any of these assumptions came from healthcare providers?
b) How did these assumptions affect how you were treated or how comfortable you felt?
20. What helped you feel that you could fully participate in your care?

Section 6: Reflections and Recommendations

21. Looking back, what changes – if any - would you like rehabilitation providers or clinics to make to help care feel safer and more inclusive?
a) Are there things they should keep doing?
b) Are there things they should do more of?
c) Are there things they should do less of or stop doing?
d) Are there things they should do differently?
22. What advice would you give to someone designing a rehabilitation program for people from diverse backgrounds?
23. Is there anything important about your experience that we haven’t talked about yet?

Section 7: Closing remarks

That brings us to the end of our conversation today. Before we finish, is there anything else you’d like to add—anything we haven’t talked about that feels important for you to share about your experience with rehabilitation care?

Thank you so much for taking the time to participate and for sharing your thoughts and experiences so openly. What you’ve shared today will help us better understand how rehabilitation services in Canada can become more inclusive, respectful, and culturally safe for everyone.

If today’s discussion brought up any difficult memories or emotions, please take care of yourself and reach out for support if you need it. This might include talking with a trusted friend, family member, Elder, or health-care provider.

You may also access free, confidential mental health and substance use support through Wellness Together Canada, which offers online resources, self-guided tools, and counselling. If you are in immediate danger or require urgent assistance, please contact local emergency services.

I’ll also share this information in the chat, and it is included in the consent form you received earlier. I’ll give you a moment if you’d like to take note of it.

***Invitation for participating in Phase 2:***

As part of this project, we’ll soon be forming a Steering Committee for the next phase of the study. The committee will bring together patients, clinicians, and community members to help co-design practical strategies that improve rehabilitation equity and cultural safety.

Would you be interested in being contacted about this opportunity? If so, we can record your interest and follow up with more details later.

Once our findings are summarized, we’ll share a plain-language summary with participants who wish to receive one.

Thank you again for your valuable contribution to this project.
